# The SGLT2 inhibitor Empagliflozin promotes post-stroke functional recovery in diabetic mice

**DOI:** 10.1186/s12933-024-02174-6

**Published:** 2024-02-29

**Authors:** Ellen Vercalsteren, Dimitra Karampatsi, Carolina Buizza, Thomas Nyström, Thomas Klein, Gesine Paul, Cesare Patrone, Vladimer Darsalia

**Affiliations:** 1 NeuroCardioMetabol Group, Department of Clinical Science and Education, Södersjukhuset, Internal Medicine, Karolinska Institutet, 118 83 Stockholm, Sweden; 2 Boehringer Ingelheim Pharma GmbH & Co. KG, Biberach, Germany; 3 Translational Neurology Group, Department of Clinical Science, Wallenberg Neuroscience Center and Wallenberg Center for Molecular Medicine, Lund University, Lund, Sweden

**Keywords:** Diabetes, Empagliflozin, Mouse, Sodium-glucose cotransporter 2 inhibitors, Stroke recovery

## Abstract

Type-2 diabetes (T2D) worsens stroke recovery, amplifying post-stroke disabilities. Currently, there are no therapies targeting this important clinical problem. Sodium-glucose cotransporter 2 inhibitors (SGLT2i) are potent anti-diabetic drugs that also efficiently reduce cardiovascular death and heart failure. In addition, SGLT2i facilitate several processes implicated in stroke recovery. However, the potential efficacy of SGLT2i to improve stroke recovery in T2D has not been investigated. Therefore, we determined whether a post-stroke intervention with the SGLT2i Empagliflozin could improve stroke recovery in T2D mice. T2D was induced in C57BL6J mice by 8 months of high-fat diet feeding. Hereafter, animals were subjected to transient middle cerebral artery occlusion and treated with vehicle or the SGLTi Empagliflozin (10 mg/kg/day) starting from 3 days after stroke. A similar study in non diabetic mice was also conducted. Stroke recovery was assessed using the forepaw grip strength test. To identify potential mechanisms involved in the Empagliflozin-mediated effects, several metabolic parameters were assessed. Additionally, neuronal survival, neuroinflammation, neurogenesis and cerebral vascularization were analyzed using immunohistochemistry/quantitative microscopy. Empagliflozin significantly improved stroke recovery in T2D but not in non-diabetic mice. Improvement of functional recovery was associated with lowered glycemia, increased serum levels of fibroblast growth factor-21 (FGF-21), and the normalization of T2D-induced aberration of parenchymal pericyte density. The global T2D-epidemic and the fact that T2D is a major risk factor for stroke are drastically increasing the number of people in need of efficacious therapies to improve stroke recovery. Our data provide a strong incentive for the potential use of SGLT2i for the treatment of post-stroke *sequelae* in T2D.

**Supplementary Information:**

The online version contains supplementary material available at 10.1186/s12933-024-02174-6.

## Introduction

Stroke is the third-leading cause of death and disability worldwide [1]. About 30% of ischemic stroke patients have diagnosed type 2 diabetes mellitus (T2D) [2], which is an established predictor of poor functional outcome [3, 4] and hampered recovery after stroke [5–7], thereby further amplifying the global disability burden. Although both pharmacological and lifestyle change strategies can reduce stroke risk in T2D [8–11], there are currently no effective therapies targeting impaired post-stroke recovery, emphasizing the necessity for new pharmacological treatments.

Hyperglycemia during acute ischemic stroke is an independent predictor of worsened post-stroke recovery [7, 12, 13]. However, intensive interventions targeting acute hyperglycemia did not result in improved functional outcome [14], and clinical studies focused on chronic post-stroke hyperglycemia regulation are lacking. Recently, pre-clinical studies from our group have demonstrated that the normalization of hyperglycemia by glucagon-like receptor 1 (GLP-1R) activation or by dipeptidyl peptidase-4 (DPP-4) inhibition in the chronic, post-acute phase after stroke was associated with improved post-stroke functional recovery in obese/diabetic mice [15, 16]**.** However, in these studies, attenuation of hyperglycemia was accompanied by the normalization of insulin resistance and an overall improvement of glucose metabolism, making it impossible to determine whether the chronic regulation of hyperglycemia per se bears therapeutic value. Additionally, the efficacy mediated by direct neurotrophic properties of GLP-1R agonists and DPP-4 inhibition independently from metabolic regulation cannot be excluded.

Sodium-glucose cotransporter-2 inhibitors (SGLT2i) are emerging anti-diabetic drugs that normalize hyperglycemia by blocking renal proximal tubular glucose reabsorption [17]. Furthermore, these drugs offer a broad range of beneficial effects beyond glycemic control, such as a reduction of weight [18] and of TD2-induced inflammation [19, 20]. Moreover, SGLT2i decrease the risk of hypoglycemic events [21] and exert beneficial effects on the cardiovascular system, not only by lowering hypertension [22] and ameliorating endothelial dysfunction [23, 24], but also by significantly improving cardiovascular outcomes [25, 26]. More specifically, in the EMPA-REG OUTCOME trial, the SGLT2i Empagliflozin exhibited significant cardiovascular benefits independently of HbA1c levels [27], stemming the international recommendations for T2D patients with cardiovascular disease to receive SGLT2i treatment in addition to metformin, regardless of baseline HbA1c levels [28]. Even though the effects of Empagliflozin on stroke risk were neutral [27], SGLT2i could improve post-stroke recovery in T2D due to their potent anti-glycemic effects as well as their impact on several processes implicated in stroke recovery. Indeed, recent literature demonstrated that a pre-stroke treatment with SGLT2i induces ischemic tolerance after stroke [29]. Moreover, SGLT2i can positively impact brain metabolism, even in non-diabetic conditions, as evidenced by both pre-clinical [30–32], and clinical studies [33].

Because of the above reported effects to modulate important processes involved in stroke recovery, and the well-known effects on attenuation of hyperglycemia, we hypothesized that SGLT2 inhibition could play a beneficial role in stroke recovery in T2D. Therefore, the aim of this study was to determine in a clinically relevant murine model of T2D and stroke whether the SGLT2i Empagliflozin improves post-stroke recovery when administered chronically in the post-stroke recovery phase. We also investigated whether potential recovery effects of Empagliflozin were associated with the regulation of glycemia and/or affected other factors involved in stroke recovery, i.e. fibroblast growth factor 21 (FGF-21) [34, 35], increased production of ketone bodies [36, 37], stroke-induced neurogenesis [38, 39], neuroinflammation [40] and post-stroke neovascularization [41].

## Materials and methods

### Animals

Eighty C57BL/6JRj mice (Janvier Labs, France) were used in this study. Mice were housed in environmentally controlled conditions (22 ± 0.5 °C, 12/12 h light/dark cycle with ad libitum access to food and water). The mice were kept under pathogen free conditions in type III size individually ventilated cages with wood chip bedding and nest material.

### Sample size calculation

Group sizes were determined based on ≈ 20% effect size between groups in functional recovery with α = 0.05 and a statistical power of 90%. Standard deviation used in sample size calculation was obtained from pilot experiments. The analyses suggested the sample size of minimum n = 5 per group. However, after taking into consideration the success rate of stroke surgery, mortality and likelihood of statistical outliers, the experimental groups were set at n = 10–15 each.

### Experimental design

#### Diabetic study

Starting at four weeks of age, mice were kept on either standard laboratory chow (n = 20, hereafter referred to as non-T2D group) or high fat diet (HFD; n = 40, 60% energy from saturated fat, hereafter referred to as T2D) for 8 months. Obesity and T2D were confirmed by a body weight increase > 20%, fasting glucose levels > 7 mmol/L, hyperinsulinemia, and decreased insulin sensitivity. Then, mice were subjected to transient middle cerebral artery occlusion (tMCAO) (n = 15 for non-T2D and n = 30 for T2D) or sham surgery (n = 5 for non-T2D and n = 10 for T2D). After tMCAO, all T2D mice were switched to SD, to reflect the clinical situation of a balanced post-stroke diet. 5 animals in the T2D group and 3 in the non-T2D group were euthanized shortly after tMCAO because the humane endpoint was reached.

**Fig. 1:**
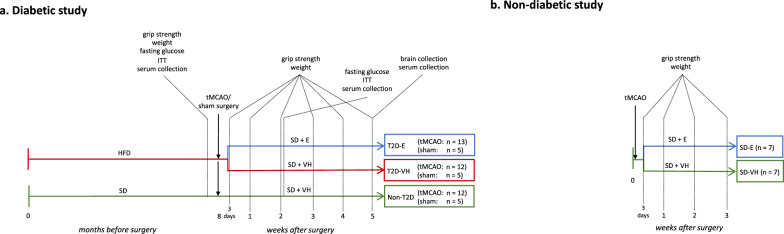
Experimental design of the studies. **a** 4-week-old male C57BL6/J mice were fed for 8 months with SD or HFD. Stroke was then induced experimentally by 30 min tMCAO and the mice on HFD were then changed to SD for the entire duration of the recovery phase. Three days after stroke, T2D mice were randomized in two groups: a group receiving 10 mg/kg/day Empagliflozin and a VH-group. During the recovery phase, behavioral tests were performed once weekly for 5 weeks. Serum was collected before stroke and at two and five weeks after stroke. The metabolic state of the animals was characterized before stroke to confirm T2D, and at 2 weeks after stroke to confirm efficacy of Empagliflozin treatment. At 5 weeks after stroke, mice were sacrificed to collect brains for immunohistochemistry and serum for assessment of metabolic parameters. **b** 3-month-old mice were subjected to tMCAO surgery with a 30 min occlusion. Three days after tMCAO, mice were randomized in 2 groups: a group receiving 10 mg/kg/day Empagliflozin and a VH-group. Behavioral tests were performed once weekly for 3 weeks. HFD = high-fat diet, SD = standard diet, ITT = insulin tolerance test, E = 10 mg/kg/day Empagliflozin p.o., VH = vehicle (0.5% methylcellulose), tMCAO = transient middle cerebral artery occlusion

Three days after stroke, the remaining T2D mice were randomized in two experimental groups and per orally treated daily with vehicle (0.5% methylcellulose solution, n = 12, hereafter referred to as T2D-VH) or the SGLT2i Empagliflozin (Boehringer-Ingelheim, Germany) (n = 13, 10 mg/kg of body weight, hereafter referred to as T2D-E). We specifically chose this delayed treatment to rule out potential acute neuroprotective effects mediated by Empagliflozin. Non-T2D animals were also treated with vehicle starting 3 days after stroke. Sham-operated animals were also randomized to either vehicle treatment (n = 5 for non-T2D and T2D-VH) or Empagliflozin treatment (n = 5). Forelimb sensorimotor function (Forelimb grip test, see below) was measured weekly for 5 weeks (timepoint where non-T2D mice were fully recovered). Then, all mice were sacrificed, and brains and serum samples were collected for analysis. See Fig. 1a for the experimental design.

#### Non-diabetic study

In this experiment, 20 adult male C57BL/6 J mice were subjected to tMCAO surgery. Shortly after tMCAO, 6 mice were euthanized because the humane endpoint was reached. Three days after stroke, mice were randomized in two experimental groups and per orally treated daily with either vehicle (0,5% methylcellulose solution, n = 7, hereafter referred to as SD-VH) or the SGLT2i Empagliflozin (10 mg/kg of body weight, n = 7, hereafter referred to as SD-E). Forelimb sensorimotor function was measured weekly during 3 weeks (timepoint where SD-VH mice fully recovered). See Fig. 1b for the experimental design.

### Transient middle cerebral artery occlusion

Stroke was induced by tMCAO using the intraluminal filament technique as described previously [42]. Briefly, mice were anesthetized by inhalation of 3% isoflurane and throughout surgery, anesthesia was maintained by 1.5% isoflurane. Using a heated pad with feedback from a thermometer, body temperature of animals was kept at 37–38 °C. Left external (ECA) and internal (ICA) carotid arteries were exposed and a 7–0 silicone-coated monofilament (total diameter 0.17–0.18 mm) was inserted into the ICA until the origin of the MCA was blocked. The occluding filament was removed after 30 min. Cerebral blood flow in the vicinity of MCA was monitored by Laser Doppler Blood Flow Monitor (Moor Instruments Ltd, UK), and no differences between the groups were observed (data not shown). Stroke induction was considered unsuccessful when the occluding filament could not be advanced within the internal carotid artery beyond 7–8 mm from the carotid bifurcation, or if mice lacked symptoms of neurological impairment based on the neurological severity score [43]. After surgery, all mice were given analgesic (Carprofen, 5 mg/kg) and soft food.

### Fasting glycemia and ITT

Fasting glycemia was measured after an overnight (ON) fasting via blood from a tail tip puncture and a glucometer. For insulin tolerance tests (ITT), mice were fasted for 2 h. Hereafter, baseline glucose levels were measured. Then, mice were injected intraperitoneally (i.p.) with 0.5 U/kg human insulin and blood glucose levels were measured at 15, 30, 45, 60, 75 and 90 min after injection. Area under the curve was computed for statistical analysis.

### Assessment of sensorimotor function

To assess sensorimotor function, forelimb grip strength was tested as previously described [44]. Briefly, mice were held firmly by the body and allowed to grasp the grid with the affected forepaw. Hereafter, they were dragged backwards until their grip was broken. Grip strength was measured using a grip strength meter (Harvard apparatus, MA, USA) at 3 days and 1–5 weeks after stroke induction. Ten trials were performed, and the highest value was recorded.

### Immunohistochemistry

Mice were anesthetized using an i.p. injection with an overdose of sodium pentobarbital. Hereafter, blood was collected via cardiac puncture and mice were perfused transcardially using phosphate-buffered saline (PBS) followed by a 4% ice-cold paraformaldehyde (PFA) solution. Brains were harvested and stored ON in 4% PFA at 4 °C. After 24 h of fixation, brains were transferred to PBS containing 25% sucrose and stored at 4 °C until they sank. Then, 30 μm thick coronal sections were cut using a sliding microtome, and sections were stored at − 20 °C in anti-freeze solution.

Immunohistochemical stainings were performed using the free-floating method. Briefly, sections were washed in PBS three times to remove anti-freeze solution. For visualization with 3–3′-Diaminobenzidine (DAB), quenching of endogenous peroxidases was performed by a 20 min incubation at RT in a PBS-solution containing 3% H_2_O_2_ and 10% methanol. For immunofluorescent DCX/Ki67 staining, sections were subjected to antigen retrieval by a 15 min incubation in citrate solution (pH = 6.0) at 95 °C. For immunofluorescent CD13, podocalyxin (PDXL), and NG2 stainings, sections were blocked with 5% serum with 0.25% triton-X100-PBS for 1 h at RT. For both DAB and immunofluorescent stainings, sections were incubated ON at 4 °C in PBS containing primary antibody, 3–5% normal serum and 0.25% Triton-X-100. For NeuN staining, sections were incubated in primary antibody solution for 48 h. The following primary antibodies were used: mouse anti-NeuN, a neuronal marker (1:500, #MAB377, Millipore, RRID:AB_2298772); goat anti-Iba1, a marker for microglia (1:1000, Ab5076, Abcam, RRID:AB_2220422); anti-DCX (doublecortin), a marker for migrating neuroblasts (1:200, sc-271390, Santa Cruz Biotechnology, RRID:AB_10610966); anti-Ki67, a marker for cell proliferation (1:300, ab15580, Abcam, RRID:AB_443209); goat anti-PDXL, a marker for endothelial cells (1:200, #AF1658, R&D Systems, RRID:JLD0117051); rat anti-CD13, a marker for pericytes (1:200, #MCA2183, Biorad, RRID:152021); rabbit anti-NG2, a marker for activated pericytes (1:200, #AB5320, Millipore, RRID:3517406); rabbit anti-albumin (1:200, ab19196, Abcam, RRID:GR3271594-14); rabbit anti-fibrinogen (1:400, ab27913, Abcam, RRID:GR28863-14). After incubation in primary antibody solution, sections were washed and then incubated 2 h at RT in PBS containing secondary antibody, 3–5% normal serum and 0.25% Triton-X-100. The following secondary antibodies were used: biotinylated horse anti-mouse (1:200, #BA-2000, Vector Laboratories, RRID:AB_2313581); biotinylated horse anti-goat (1:200, #BA-9500, Vector Laboratories, RRID:AB_2336123); Alexa-488 conjugated horse anti-rabbit (1:200, DI-1088, Vector Laboratories, RRID:AB_2336403); Alexa-584 conjugated horse anti-mouse (1:200, DI-2594, Vector Laboratories, RRID:AB_2336412); Alexa-488 conjugated donkey anti-rabbit (1:500, #711-545-152, JacksonImmuno Research, RRID:156009); Alexa-546 conjugated donkey anti-goat (1:500, #A11056, Invitrogen, RRID:997810); Cy5 conjugated donkey anti-rat (1:500, #712–175-150, JacksonImmuno Research, RRID:148159). For DAB-visualization, incubation with biotinylated secondary antibody was followed by incubation with avidin–biotin complex according to manufacturer’s instructions (Vectastain Elite ABC kit, Vector Laboratories), followed by visualization by DAB. For DCX/Ki67 staining, incubation with secondary antibody solution was followed by a 30 s incubation with PBS containing 1:10 000 DAPI.

### Quantitative microscopy and image analysis

#### Ischemic stroke volume assessment

Ischemic volume was measured using all serial sections containing visual ischemic damage. Briefly, NeuN-labelled sections were displayed live on a computer monitor using a 1.25 × lens. Volume of the whole contralateral, non-damaged hemisphere, and of the intact part of the ipsilateral, stroke-damaged hemisphere was determined using the Cavalieri Estimator probe (StereoInvestigator, MBF Bioscience, USA). The ischemic volume was then determined by subtracting the ipsilateral volume from the entire contralateral volume, thus adjusting for stroke-induced tissue shrinkage.

#### Assessment of neuroinflammation

The Fiji opensource image analysis software was used to evaluate Iba-1 immunoreactivity [45]. Briefly, images of Iba-1 staining in striatum were acquired at 20 × magnification using the Olympus BX40 microscope. Images were then converted into grayscale (8-bit) mode and thresholded. The lowest Iba-1 immunoreactivity in the non-T2D group was used as baseline to determine the threshold. For each hemisphere, 3 images containing > 90% of the striatum were analyzed, resulting in a total of 9 pictures analyzed per hemisphere per animal. The Iba-1^+^ area was measured and expressed as percentage of total area. Animals with an ipsilateral Iba-1 response less than 1.5-fold compared to their contralateral hemisphere were classified as non-responders and excluded from analysis.

#### Assessment of neurogenesis

Manual counting of Ki67 in the subventricular zone and of DCX in striatum was performed on three coronal brain sections using the Olympus BX40 microscope. The first section was selected based on its anatomical location along the rostral-caudal axis (approximately 1 mm from Bregma). The second and third sections were 300 and 600 μm caudal from the first section, respectively. The number of Ki67 + cells in the subventricular zone and of DCX in the striatum was manually counted in all three sections using a dry 40 × lens. All counts were performed by experimenters blinded for experimental groups.

#### Assessment of vascularization and blood–brain barrier leakage

Two brain sections were selected for assessing vascularization, and one brain section was selected for assessing blood–brain barrier (BBB) leakage. Confocal images were obtained using a Leica DMi8 confocal microscope. One to two images per section were taken at 20 × magnification from the dorsolateral and medial striatum depending on the dimension of the brain (image size: 775 μm × 775 μm; z-stack size = 10 μm; step size = 0.5 μm). The same acquisition settings were applied for each image. Immunohistochemical images were compared to the images from the NeuN staining to visualize the ischemic core, and the images from regions outside of the ischemic core were excluded. Quantification of the vascularization parameters and BBB leakage were performed on the maximum projected and automatically thresholded images using the area fraction measurement tool of Fiji open-source image analysis software [45]. The area density was expressed as the percentage of PDXL and CD13 of the total image area. Pericyte coverage of the vessels was obtained by calculating the area of the colocalizing CD13 and PDXL signals and normalizing it to the total PDXL area of the same image. Activated pericytes were identified by NG2 [46]. The area of the activated pericytes was obtained by calculating the area of the colocalizing NG2 and CD13 signals and normalizing it to the total CD13 area of the same image. The density of parenchymal pericytes was calculated on the maximum projected and automatically thresholded images by subtracting the colocalizing PDXL/CD13 pixels from the CD13 ones. For vessel length and branch counts, the maximum projected images were binarized by automatic thresholding and skeletonized, and the skeletons were analyzed using the AnalyzeSkeleton plugin [47] as previously described [48]. Extravascular fibrinogen and albumin were quantified to evaluate BBB leakage. PDXL vessels were outlined to exclude intravascular plasma proteins. Then, by applying an automatic image threshold, the area covered by extravascular fibrinogen and albumin was quantified and expressed as the percentage of the total image area using the area fraction measurement tool. The image analysis was scripted and automated using the programming language ImageJ Macro minimizing the potential for human error or bias.

### Quantification of serum insulin, FGF-21 and β-hydroxybutyrate

Serum concentrations of insulin, FGF-21 and β-hydroxybutyrate (BHB) were quantified according to manufacturer’s instructions in serum samples obtained before stroke and at 2 and 5 weeks after tMCAO (90080, CrystalChem for insulin; R&D Systems MF2100, R&D Systems for FGF-21, ab93380, Abcam for β-hydroxybutyrate).

### Data and statistical analysis

Data were checked for statistical outliers by using the ROUT method, and for normality by using the Shapiro–Wilk normality test.

*Parametric tests:* For pre- and post-stroke metabolic parameters and NeuN analysis, Brown-Forsythe and Welch ANOVA test, followed by two-stage linear step-up procedure of Benjamini, Krieger, and Yekutieli was used. For behavioral tests, two-way repeated measures ANOVA with Geisser-Greenhouse's correction followed by Dunnett T3 was used. For neuroinflammation, neurogenesis and vascular analysis, two-way repeated measures ANOVA followed by two-stage linear step-up procedure of Benjamini, Krieger, and Yekutieli was used. All data were analyzed by GraphPad Prism Version 9.0. Data are expressed as mean ± SD. *p*-values less than 0.05 were considered statistically significant.

## Results

### Empagliflozin significantly improves post-stroke functional recovery in T2D mice in association with the normalization of glycemia.

**Fig. 2:**
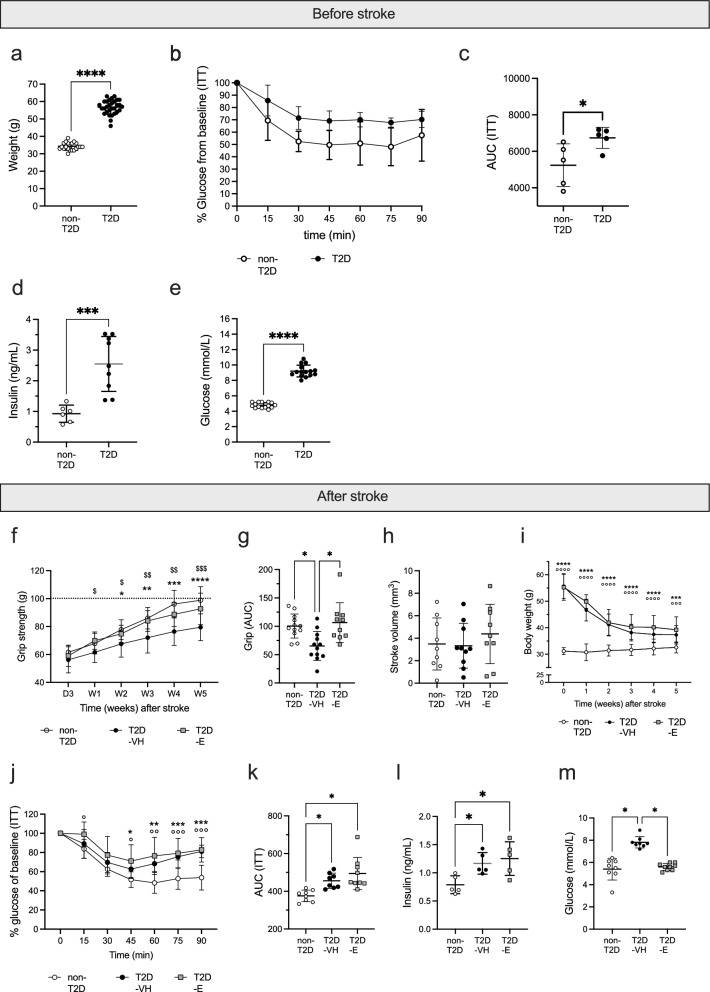
Effect of Empagliflozin treatment on metabolic parameters and functional recovery after stroke. Effect of 8 months of HFD on weight (**a**), insulin sensitivity shown as plotted curve (**b**) and area under the curve (**c**), serum insulin levels (**d**) and fasting glycemia (**e**). Forepaw grip strength after stroke shown as plotted curve (**f**) and area under the curve (**g**). Ischemic stroke volume (**h**). Body weight during stroke recovery (**i**). Insulin sensitivity, shown as plotted curve (**j**) and area under the curve (**k**), serum insulin (**l**) and fasting glycemia (**m**) at 2 weeks after stroke. Data are presented as mean ± SD. Statistical significance was calculated using two-way repeated measures ANOVA followed by Benjamini, Krieger and Yekutieli multiple comparisons test for insulin tolerance tests, forepaw grip strength and post-stroke body weight (**b**, **f**, **i**, **j**), Welch’s t-test for weight, area under the curve for ITT, plasma insulin and fasting glycemia before stroke (**a**, **c**–**e**) Brown-Forsythe and Welch’s one-way ANOVA followed by Benjamini, Krieger and Yekutieli multiple comparisons test for post-stroke area under the curve of grip and ITT, stroke volume, plasma insulin and fasting glycemia (**g**, **h**, **k**–**m**). Results were considered significant if p < 0.05. *denotes a significant difference between non-T2D and T2D-VH, °denotes a significant difference between non-T2D and T2D-E, ^$^denotes a significant difference between T2D-VH and T2D-E. * and ^$^denote p < 0.05, ** and ^$$^denote p < 0.01, ***, °°° and ^$$$^denote p < 0.001, **** and °°°°denote p < 0.0001. Sample size: **a** non-T2D n = 20, T2D n = 30; **b**, **c** n = 5 in both non-T2D and T2D; **d** non-T2D n = 6, T2D n = 9;** e** n = 15 in both non-T2D and T2D; **f**, **g** non-T2D n = 12, T2D-VH n = 12, T2D-E n = 10; **h** non-T2D n = 9, T2D-VH n = 10, T2D-E n = 9; **i** non-T2D n = 12, T2D-VH n = 12, T2D-E n = 13; **j**, **k** non-T2D n = 8, T2D-VH n = 8, T2D-E = 9; **l** non-T2D n = 5, T2D-VH n = 5, T2D-E n = 5; **m** non-T2D n = 8, T2D-VH n = 8, T2D-E n = 9

As recently published [15, 16, 39], 8 months of HFD-feeding induced obesity (Fig. 2a), decreased insulin sensitivity (Fig. 2b, c), hyperinsulinemia (Fig. 2d) and hyperglycemia (Fig. 2e).

To assess the potential efficacy of Empagliflozin to improve stroke recovery, forepaw grip strength recovery was followed up for 5 weeks after tMCAO. After tMCAO, non-T2D mice recovered fully within 5 weeks while T2D-VH mice remained significantly impaired (Fig. 2f, g). Importantly, Empagliflozin treatment completely normalized the T2D-induced worsening of stroke recovery (Fig. 2f, g). No differences in stroke volume were observed between groups (Fig. 2h), demonstrating that the improved recovery was not due to differences in infarct size mediated by Empagliflozin-induced neuroprotection.

To investigate the potential association between Empagliflozin-induced improved recovery and metabolic changes, we analyzed several metabolic parameters after stroke. In accordance with previous studies [49–51], tMCAO and subsequent switch from HFD to SD induced significant weight loss in the first two weeks after tMCAO in all T2D mice, without significant differences between T2D-VH (− 34 ± 3%) and T2D-E (− 28 ± 7%) groups (Fig. 2i). At 2 weeks after stroke, all T2D mice were still IR (Fig. 2j, k) and hyperinsulinemic (Fig. 2l), irrespective of treatment. However, T2D-E mice became normoglycemic while hyperglycemia was still present in T2D-VH mice, showing that Empagliflozin efficiently reduced hyperglycemia, but not IR after stroke in our model (Fig. 2m).

Unlike T2D mice subjected to tMCAO, T2D mice subjected to sham surgery only lost 12% of their initial body weight during the first two weeks post-diet change, resulting in differences in the metabolic state between stroke and sham mice (Additional file 1: Fig. S1a). In sham-operated animals treated with Empagliflozin, a trend (p = 0.112) towards attenuated hyperglycemia was observed, whereas no effect on insulin sensitivity was observed (Additional file 1: Fig. S1b–d).

In summary, our results demonstrate that a post-stroke treatment with Empagliflozin significantly improves post-stroke recovery, in association with the normalization of hyperglycemia.

### Improved stroke recovery by Empagliflozin is associated with increased post-stroke serum levels of FGF-21 but not BHB

**Fig. 3:**
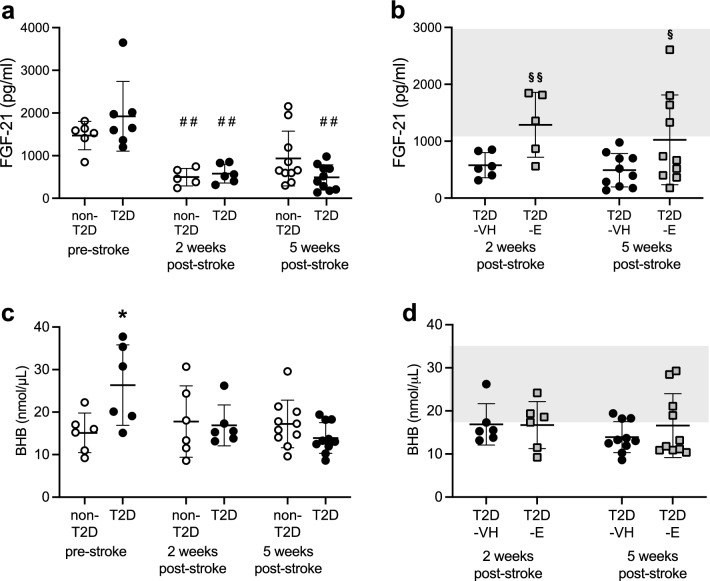
Effect of stroke and Empagliflozin treatment on serum FGF-21 and BHB concentrations. Serum fibroblast growth factor 21 (FGF-21) (**a**) and β-hydroxybutyrate (BHB) (**c**) levels before stroke and at 2 and 5 weeks after stroke in non-diabetic controls (non-T2D) and type-2 diabetic mice (T2D). Serum FGF-21 (**b**) and BHB (**d**) levels of T2D mice treated with VH (T2D-VH) and diabetic mice treated daily with 10 mg/kg Empagliflozin (T2D-E) at 2 and 5 weeks after stroke. The grey area indicates the range of pre-stroke levels of Fgf-21 (**b**) and BHB (**d**) in T2D mice. Data are presented as mean ± SD. Statistical significance was calculated using two-way repeated measures ANOVA followed by Benjamini, Krieger and Yekutieli multiple comparisons test. Results were considered significant if p < 0.05. ^§^denotes a significant difference between T2D-VH and T2D-E, ^*^denotes a significant difference between non-T2D and T2D, ^#^denotes a significant difference compared to pre-stroke in the same group. *denotes p < 0.05, **, ^§§^ and ^##^denote p < 0.01. Sample size: n = 5–10 per group. At the pre-stroke and intermediate post-stroke timepoint, each data point represents results from serum pooled from 2–3 animals

Increased FGF-21 levels have been associated with post-stroke recovery [34]. Moreover, Jiang and colleagues recently demonstrated that a therapeutic administration of FGF-21 improves post-stroke recovery in diabetic mice [35]. Since recent literature has shown that SGLT2i can increase FGF-21 levels [52], we investigated whether the improved recovery in the T2D-E group was associated with increased serum levels of FGF-21. Before stroke, no difference in FGF-21 levels between non-T2D and T2D mice was recorded (Fig. 3a). At two weeks after stroke, FGF-21 levels were significantly decreased in both non-T2D and T2D-VH mice (Fig. 3a) and remained significantly lower than pre-stroke levels in T2D-VH (p = 0.01) at five weeks post-stroke (Fig. 3a). Interestingly the post-stroke treatment with Empagliflozin resulted in a significant increase of serum FGF-21 levels, both at two and at five weeks after stroke (Fig. 3b). Taken together, these results indicate that stroke decreases serum FGF-21 levels independently of the metabolic state of the animals, and that Empagliflozin prevents this stroke-induced reduction, in association with improved recovery.

In accordance with existing literature [53], HFD-induced T2D significantly increased serum BHB-levels (Fig. 3c). After stroke, there was a trend towards a decrease in serum BHB (p = 0.198 at 2 weeks and p = 0.056 at 5 weeks after stroke) in T2D-VH mice compared to pre-stroke levels (Fig. 3c). We found no difference between T2D-VH and T2D-E mice at either 2 or 5 weeks after stroke (Fig. 3d), indicating that after stroke, SGLT2i-treatment does not upregulate ketone production in T2D mice.

### Effect of Empagliflozin treatment on post-stroke neurogenesis

**Fig. 4:**
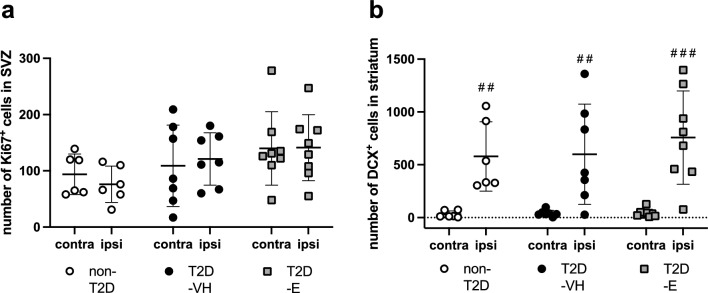
Effect of Empagliflozin on neurogenesis after stroke. Number of Ki67^+^ cells in subventricular zone (SVZ) (**a**) and number of DCX^+^ cells in striatum (**b**) of non-diabetic controls (non-T2D), diabetic mice (T2D-VH) and diabetic mice treated with Empagliflozin (T2D-E) after stroke. Data are presented as mean ± SD. Statistical significance was calculated using two-way ANOVA followed by Benjamini, Krieger and Yekutieli multiple comparisons test. Results were considered statistically significant if p < 0.05. ^#^denotes a difference between the contralateral and ipsilateral hemisphere within the same group. ^##^denotes p < 0.01, ^###^denotes p < 0.001. non-T2D n = 6, T2D-VH n = 7, T2D-E n = 8

Stroke-induced neurogenesis has been associated with improved stroke recovery (reviewed in [39]. Therefore, we next assessed whether improved functional recovery by Empagliflozin after stroke was associated with the regulation of this process. Neural stem cell proliferation and neuroblast formation were analyzed by quantifying Ki67^+^ cells in the SVZ and DCX^+^ neuroblasts in the striatum, respectively. No differences in Ki67^+^ cells were recorded between groups in the SVZ (Fig. 4a). In accordance with existing literature [51], stroke induced a significant increase in DCX^+^ cells in the ipsilateral, stroke-damaged striatum in all three groups (Fig. 4b). However, there was no difference in the number of DCX^+^ cells between the groups (Fig. 4b), suggesting that improved stroke recovery in the T2D-E group was not due to increased neurogenesis.

### Effect of Empagliflozin treatment on T2D-induced Iba-1 immunoreactivity

**Fig. 5:**
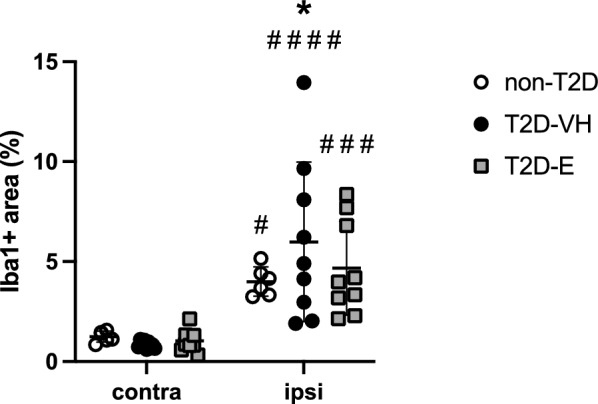
Effect of Empagliflozin on neuroinflammation after stroke. Iba-1 expression in striatum of non-diabetic controls (non-T2D), diabetic mice (T2D-VH) and diabetic mice treated with Empagliflozin (T2D-E) after stroke. Data are presented as mean ± SD. Statistical significance was calculated using two-way ANOVA followed by Benjamini, Krieger and Yekutieli multiple comparisons test. Results were considered statistically significant if p < 0.05. *denotes a significant difference between non-T2D and T2D-VH in the same hemisphere, ^#^denotes a significant difference between the contralateral and ipsilateral hemisphere within the same group. ^#^ and *denote p < 0.05, ^###^denotes p < 0.001 and ^####^denotes p < 0.0001. non-T2D n = 6, T2D-VH n = 9, T2D-E n = 9

To evaluate stroke-induced neuroinflammation, we quantified Iba-1 immunoreactivity in ipsilateral, stroke-damaged striatum *versus* the intact contralateral hemisphere. Stroke induced a significant upregulation of Iba-1 in ipsilateral striatum compared to contralateral in all three groups. Notably, this increase was significantly higher in T2D-VH mice than in non-T2D mice (Fig. 5). However, Empagliflozin treatment did not significantly decrease Iba-1 immunoreactivity compared to T2D-VH animals, although a trend was observed (p = 0.103) (Fig. 5). Moreover, we observed no apparent morphological differences of striatal microglia between groups. In sham-operated animals, T2D upregulated striatal Iba-1 compared to non-T2D controls, but no differences between groups were detected between sham-T2D-VH and sham-T2D-E animals (Additional file 1: Fig. S2). Taken together, these data indicate that, at least at 5 weeks after stroke, improved stroke recovery in Empagliflozin treated animals is likely not associated with attenuated post-stroke Iba-1 immunoreactivity.

### Effect of Empagliflozin treatment on post-stroke neovascularization

To investigate whether Empagliflozin treatment has an impact on the vascular system after stroke, we evaluated cerebral vascular changes in terms of vessel (PDXL^+^), total pericyte density (CD13^+^) and coverage (CD13^+^/PDXL^+^ ratio), vessel length and branching, markers of pericyte activation (CD13^+^/NG2^+^), and BBB leakage by assessing extravascular albumin and fibrinogen.

**Fig. 6:**
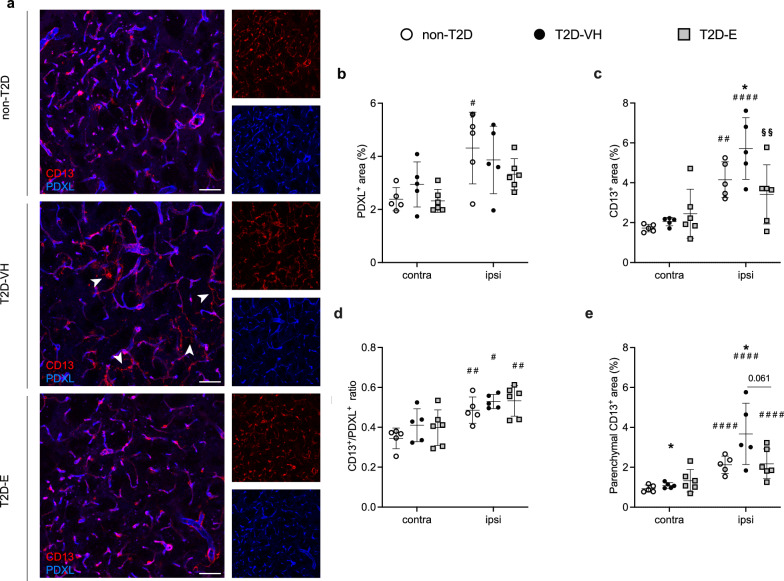
Effect of Empagliflozin on vascularization after stroke. Confocal images (**A**) showing the expression in the striatum of non-diabetic controls (non-T2D), diabetic mice (T2D-VH) and diabetic mice treated with Empagliflozin (T2D-E) after stroke of CD13 (red) and PDXL (blue) evaluating vessel density (**B**), pericyte density (**C**), pericyte coverage (**D**) and parenchymal pericyte density (**E**). White arrows indicate the pericytes that are not associated with the vessels. Data are presented as mean ± SD. Statistical significance was calculated using two-way ANOVA followed by Benjamini, Krieger and Yekutieli multiple comparisons test. Results were considered statistically significant if p < 0.05. *denotes a difference between non-T2D and T2D-VH, ^§^denotes a difference between T2D-VH and T2D-E, ^#^denotes a difference between contralateral and ipsilateral hemisphere within the same group. Scale bar = 50 μm. non-T2D n = 5, T2D-VH n = 5, T2D-E n = 6

In non-T2D mice subjected to stroke, the injury significantly increased vascular density, total pericyte density, parenchymal pericyte density, pericyte coverage, and density of activated pericytes in the ipsilateral striatum compared to the contralateral, indicating post-stroke angiogenesis (Fig. 6a–e and Additional file 1: Fig. S3). In T2D-VH stroke mice, total and parenchymal pericyte density, coverage and activation were increased in the ipsilateral striatum (Fig. 6a–e, Additional file 1: Fig. S3). In T2D-E animals, stroke upregulated parenchymal pericyte density and coverage in the ipsilateral striatum compared to the contralateral hemisphere (Fig. 6a–e and Additional file 1: Fig. S3).

When comparing ipsilateral hemispheres between groups, two-way ANOVA revealed a significant increase in the total pericyte density in the T2D-VH group compared to the non-T2D group, which was normalized by Empagliflozin treatment (Fig. 6b). Interestingly, groups did not differ in vascular density (Fig. 6c) and had similar pericyte coverage of the vessels (Fig. 6d), implicating that the increased overall pericyte density observed in the T2D-VH group is due to pericytes located in the parenchyma. Indeed, we observed a significant increase in parenchymal pericyte density in the T2D-VH group compared to the non-T2D group (Fig. 6e). Moreover, there was a strong trend towards a decreased parenchymal pericyte density in the T2D-E group compared to the T2D-VH group (p = 0.061), and no difference was detected between non-T2D and T2D-E animals (Fig. 6e). No differences were seen between the three experimental groups within the contralateral striatum, with the exception of increased parenchymal pericyte density in the T2D-VH *vs*. non-T2D groups, indicating that T2D impacts the balance between perivascular and parenchymal pericytes (Fig. 6a–e). Pericyte activation was similarly activated in all groups after stroke (Additional file 1: Fig. S3). Taken together, these data indicate that T2D alters parenchymal pericyte density, and that Empagliflozin-treatment can normalize this effect.

To assess BBB integrity, we examined the presence of plasma proteins in the brain parenchyma analyzing two different molecular sizes, albumin (~ 65 KDa) and fibrinogen (~ 340 KDa). We observed no significant differences in albumin or fibrinogen extravasation between the groups, (Additional file 1: Fig. S4). In sham-operated animals, treatment with Empagliflozin led to a significant increase in total and parenchymal pericyte density and coverage, whereas no differences were found in BBB integrity between groups (Additional file 1: Fig. S5).

### Empagliflozin does not improve post-stroke functional recovery in non-T2D mice

**Fig. 7:**
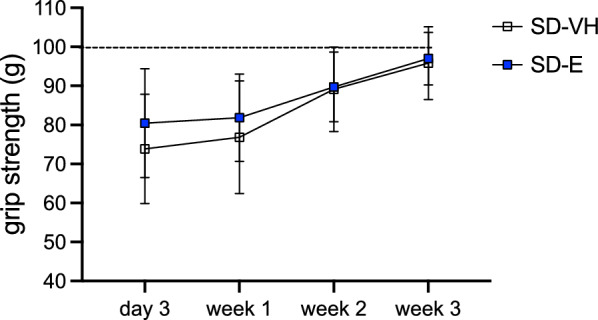
The effect of Empagliflozin on functional recovery after stroke in non-T2D mice. Forepaw grip strength of non-T2D mice treated with vehicle (SD-VH) or daily treatment with 10 mg/kg Empagliflozin p.o. (SD-E) after stroke shown as plotted curve. Data are presented as mean ± SD. Statistical significance was calculated using two-way repeated measures ANOVA followed by Benjamini, Krieger and Yekutieli multiple comparisons test and results were considered significant when p < 0.05. SD-VH n = 7, SD-E n = 7

We next determined whether a post-stroke intervention with Empagliflozin could improve recovery independently of its glycemia-regulating properties. Non-T2D mice were treated daily with either VH or Empagliflozin starting from 3 days after stroke until Empagliflozin-treated mice fully recovered (Exp. design Fig. 1b). There was no difference in forepaw grip strength between the groups (Fig. 7), indicating that the improved stroke recovery induced by Empagliflozin in the diabetic study was likely mediated by the anti-T2D properties of Empagliflozin.

## Discussion

The aim of this study was to determine whether the SGLT2i Empagliflozin improves post-stroke recovery in T2D when administered chronically in the post-stroke recovery phase. We demonstrated that Empagliflozin significantly improves stroke recovery, and this effect occurs in association with attenuated hyperglycemia, elevated serum FGF-21 levels and normalization in parenchymal pericyte density in the infarct core. Five weeks after stroke, Empagliflozin-treatment did not affect the production of ketone bodies, post-stroke neurogenesis or inflammation. Moreover, we showed that in non-T2D mice, a post-stroke intervention with Empagliflozin had no effect on stroke recovery.

Our experimental design was conceived with the idea to prove potential recovery effects mediated by Empagliflozin independently from acute neuroprotection which has recently been demonstrated [29, 54]. Therefore, we initiated the treatment only 3 days post-stroke. Indeed, Empagliflozin treatment after stroke improved recovery without affecting infarct size, thus excluding acute neuroprotective effects of Empaglifozin in our study.

Recent studies have demonstrated that SGLT2i can pass the BBB [55–57] and can boost neuronal activity [58–62]. However, since Empagliflozin-treatment did not improve stroke recovery in the non-T2D study, it is highly likely that the recovery-effects observed in the diabetic study were mediated by the anti-diabetic properties of the drug and were not due to direct brain effects. Indeed, previous work from our group has shown that a prolonged treatment with the GLP1- agonist Exendin-4 [49] and the DPP-4 inhibitor Linagliptin [50] initiated after stroke, improved stroke recovery in association with normalized glucose metabolism. Interestingly, unlike Exendin-4 and Linagliptin that affect both hyperglycemia and insulin resistance, Empagliflozin specifically attenuated hyperglycemia without affecting insulin sensitivity, indicating that sustained glycemic control post-stroke might be sufficient to improve stroke recovery in diabetes.

Post-stroke recovery effects might be associated with the regulation of stroke-induced adult neurogenesis [63] and/or neuroinflammation [64]. We have shown in previous studies that the DPP-4 inhibitor Linagliptin enhances the number of stroke-induced DCX^+^ neuroblasts in association with improved stroke recovery, even though T2D per se did not affect this cellular process [50, 65]. However, in the present study, we found no effect of Empagliflozin on DCX^+^ neuroblasts, suggesting that SGLTi and DPP-4 inhibitors exert their beneficial effects on stroke recovery via different mechanisms of action.

Stroke-induced neuroinflammation is a complicated and multifaceted, yet vital process for stroke recovery [40, 66]. Diabetes disrupts the intricate balance between pro- and anti-inflammatory responses after stroke, thereby hampering stroke recovery [67]. We have recently demonstrated exacerbated neuroinflammation in the post-stroke recovery phase of T2D mice after prolonged HFD feeding [49–51] as well as the effect of different T2D drugs to counteract this effect [49, 50]. Since SGLT2i have been shown to dampen exacerbated neuroinflammation induced by T2D both in vitro and in vivo [68–70], we hypothesized that Empagliflozin might dampen the neuroinflammatory process in the recovery phase after stroke. As expected, we found that stroke increased ipsilateral microglia-infiltration which was significantly higher in the T2D-VH group compared to non-T2D controls. However, Empagliflozin treatment did not affect the amount of Iba-1^+^ microglia in the ipsilateral hemisphere, suggesting that the beneficial effect of Empagliflozin on stroke recovery was not due to attenuated T2D-induced inflammation, at least not at the 5 weeks post-stroke timepoint, when the mice were sacrificed.

The positive effect of ketone bodies on the brain is well known [37, 71, 72]. Since SGLT2i increase ketone production [36, 73, 74] and this mechanism has been proposed to play a role in cardiovascular outcome [75], we hypothesized that this mechanism could be also involved in Empagliflozin-improved stroke recovery. However, serum BHB-levels were not increased in the obese/T2D-E group after stroke. The increased ketone-production upon SGLT2i-treatment is modest [53, 76], suggesting that the effects of Empagliflozin on serum BHB might have been masked by the pronounced tMCAO-induced weight loss inherent to our T2D/stroke model. In addition, ketone bodies production following treatment with SGLT-2i are much more pronounced in T2D patients vs impaired fasted glucose individuals, and our HFD animal model resembles more a mild T2D [77]. Taken together, this suggests that it is unlikely that the improved post-stroke recovery by Empagliflozin occurs via increased ketone production in this study.

Here, we show that the improved stroke recovery in the T2D-E group was associated with elevated post-stroke FGF-21 serum levels.

FGF-21 is an important regulator of glucose and lipid metabolism, that has also been shown to have beneficial effects on stroke recovery [78–80]. In accordance with a recent study by Wang et al., we found that FGF-21-levels were reduced after stroke in the non-diabetic mice [81]. Moreover, we demonstrated that this FGF-21 reduction is not affected by T2D. Interestingly, Empagliflozin treatment inhibited this stroke-induced decrease both at 2 and 5 weeks after stroke. This is in line with existing literature indicating that SGLT2i treatment increases plasma FGF-21 levels [52, 82, 83]. Interestingly, FGF-21 has been positively associated with improved stroke recovery, both in pre-clinical and clinical studies [34, 84]. Furthermore, an intervention with recombinant FGF-21, either acutely or in the chronic phase after stroke, significantly improved recovery in diabetic mice [78, 79, 85]. Therefore, although speculative, our results highlight FGF-21 as a potential mechanism for improved stroke recovery mediated by SGLT2i treatment.

Efficient post-stroke angiogenesis and vascular remodeling are crucial for effective stroke recovery [86]. T2D disrupts these processes, thereby impairing stroke recovery [41], whereas anti-diabetic treatments can revert aberrant vascular remodeling, thus restoring BBB-integrity [87]. Moreover, we recently showed that the post-stroke administration of the GLP-1R agonist Exendin-4 restored vascular remodeling after stroke, in association with improved recovery [49]. Emerging evidence indicates beneficial effects of SGLT2i on vascularization [23, 24]. Indeed, SGLT2i improve remodeling of the neurovascular unit in T2D [88] and stroke [29]. Similar effects were observed in diabetic mice with a post-stroke administration of recombinant FGF-21 [35]. Therefore, we investigated the potential role of Empagliflozin on post-stroke vascular remodeling. Our results show that a post-stroke intervention with Empagliflozin normalizes parenchymal pericyte density in the infarct core in T2D mice.

Following stroke, angiogenesis and vascular remodeling are essential to restore the ischemic tissue with oxygen and nutrients and therefore favor the recovery of the tissue after stroke [89]. In general, enhanced tissue perfusion and increased vessel density are beneficial in recovery; but at the same time, extended angiogenesis might be accompanied by BBB leakage [90–92]. While we observed clear stroke-induced effects when comparing contralateral and ipsilateral hemispheres, diabetes did not determine relevant effects in terms of vascularization, except for an increase in pericyte density which, interestingly, was normalized by Empagliflozin treatment. The changes in pericyte density were not complemented by alterations in vessel density, pericyte coverage or pericyte activation and were in accordance with the fact that BBB leakage was also not detected, perhaps due to the late time point selected for the analysis after ischemic injury. Since T2D was associated with a higher pericyte density which was not reflected in increased vascular coverage, we assessed the density of parenchymal pericytes. Previous studies in literature report that following a stroke, platelet-derived-growth-factor beta (PDGFRß) positive cells (a marker of pericytes) within the infarct core migrate away from the blood vessels into the parenchyma [93–95]. It has been proposed that these parenchymal PDGFRß^+^ cells are involved in the formation of the fibrotic scar following stroke by depositing extracellular matrix proteins [96]. T2D increased parenchymal pericytes density compared to non-T2D controls, and treatment with Empagliflozin normalized this effect. Therefore, our data suggest that Empagliflozin treatment might prevent or resolve this T2D-induced shift in the location of the pericytes from a perivascular to a parenchymal location. The functional significance of this phenomenon is unclear, but a relation to the improved functional recovery cannot be ruled out.

SGLT2i efficiently attenuate T2D-induced cardiac fibrosis and oxidative stress, thereby improving cardiac function, prompting these drugs to be implemented for heart failure treatment [97–99]. Recently, diabetes-induced ROS-production and senescence have been proposed as cellular mechanisms behind this impaired cardiac function [100, 101]. Interestingly, ischemic stroke induces increased ROS-production and senescence in the brain [102–104], and interventions to decrease ROS and senescence can improve neurological function after stroke [105–107]. Since SGLT2i have been shown to attenuate T2D-induced senescence and ROS production [108–110], this could be an additional cellular mechanism behind the improved stroke recovery that should be investigated in future studies.

There are limitations to the present study that need to be acknowledged. First, an additional timepoint to perform IHC studies would have helped to more thoroughly characterize cellular processes involved in stroke recovery such as neuroinflammation and neurogenesis. In addition, although we showed a positive association between Empagliflozin-induced improvement in stroke recovery and increased FGF-21 levels, we did not address whether this is indeed a causative mechanism of improved functional recovery. In this respect, new studies using Empagliflozin in the presence of FGF-21 antagonists [111] will be needed. Finally, our study demonstrates the efficacy of a post-stroke intervention with SGLT2i to improve recovery in T2D. Although these data are encouraging, they do not provide insight in the potential benefit of a pre-stroke intervention with SGLT2i on stroke recovery in T2D. Of interest in this respect was the recent study of Takashima and colleagues, demonstrating improved neurological recovery with a pre-stroke SGLT2i-intervention in non-diabetic mice [29].

Based on the mechanistic action of SGLT2i in enhancing glucose excretion, which is compensated by an increased hepatic glucose production, we are currently establishing a suitable experimental design to test a pre-stroke intervention with SGLT2i in HFD animals. In particular, the catabolic status of the animals during weight loss after stroke, together with a shift in diet after tMCAO that might impact ketone body generation will also need to be taken into account.

## Conclusions

Our study shows that a post-stroke intervention with the SGLT2i Empagliflozin improves stroke recovery in T2D mice. Moreover, it has recently been shown that SGLT2i-treatment, both in normal and hyperglycemic rodent models [54, 112, 113], acutely after stroke significantly decreased infarct size and ameliorated neurobehavioral outcome after stroke. Taken together, these data demonstrate additional advantage of SGLT2i-based therapies for patients with T2D, not only to treat diabetes and to reduce associated co-morbidities [28], but potentially also to improve stroke recovery.

### Supplementary Information

**Additional file 1:** **Figure S1.** Effect of Empagliflozin-treatment on the metabolic profile of sham-operated animals. **Figure S2.** Effect of Empagliflozin-treatment on striatal Iba1 expression in sham-operated animals. **Figure S3.** Effect of Empagliflozin-treatment on pericyte activation. **Figure S4.** Effect of Empagliflozin-treatment on BBB leakage and vascularization after stroke. **Figure S5.** Effect of Empagliflozin-treatment on BBB leakage and vascularisation in sham-operated animals. 

## Data Availability

The data that support the findings of this study are available from the corresponding authors upon reasonable request.

## Abbreviations

BBB: Blood–brain barrier
BHB: β-Hydroxybutyrate
DAB: 3–3′-Diaminobenzidine
DCX: Doublecortin
DPP4: Dipeptidyl-peptidase 4
FGF-21: Fibroblast growth factor 21
GLP-1R: Glucagon-like peptide 1 receptor
HFD: High-fat diet
IHC: Immunohistochemistry
ITT: Insulin tolerance test
ON: Overnight
PBS: Phosphate-buffered saline
PDGFRβ: Platelet-derived growth factor beta
PDXL: Podocalyxin
PFA: Paraformaldehyde
RT: Room temperature
SGLT2i: Sodium glucose co-transporter inhibitor
SVZ: Subventricular zone
T2D: Type-2 diabetes
tMCAO: Transient middle cerebral artery occlusion
VH: Vehicle
