# Supplementary material for: The SGLT2 inhibitor Empagliflozin promotes post-stroke functional recovery in diabetic mice
Source: Cardiovasc Diabetol. 2024 Feb 29;23:88. doi: 10.1186/s12933-024-02174-6 (PMC10905950; doi:10.1186/s12933-024-02174-6)
Supplement: Supplementary file 1 — Additional file 1: Figure S1. Effect of Empagliflozin-treatment on the metabolic profile of sham-operated animals. Figure S2. Effect of Empagliflozin-treatment on striatal Iba1 expression in sham-operated animals. Figure S3. Effect of Empagliflozin-treatment on pericyte activation. Figure S4. Effect of Empagliflozin-treatment on BBB leakage and vascularization after stroke. Figure S5. Effect of Empagliflozin-treatment on BBB leakage and vascularisation in sham-operated animals. [file 12933_2024_2174_MOESM1_ESM.docx]

**ADDITIONAL FILE 1: FIGURES**

**Fig S1: Effect of Empagliflozin-treatment on the metabolic profile of sham-operated animals.** Body weight (a), fasting glycemia (b), and insulin sensitivity shown as plotted curve (c) and area under the curve (d) for non-diabetic controls (sham non-T2D), diabetic animals treated with vehicle (sham T2D-VH) and diabetic animals treated daily with 10 mg/kg Empagliflozin perioral at 2 weeks after sham surgery. N = 5 per group. Data are presented as mean ± SD. Statistical significance was calculated using Brown-Forsythe and Welch one way ANOVA followed by a *post hoc* multiple comparisons test using the two-stage step-up method of Benjamini, Krieger and Yekutieli (b, d) or two-way repeated measures ANOVA followed by Benjamini, Krieger and Yekutieli multiple comparisons test (a, c). Results were considered significant if p < 0.05. * denotes a significant difference between non-T2D and T2D-VH and ° denotes a significant difference between non-T2D and T2D-E.

**Fig S2: Effect of Empagliflozin-treatment on striatal Iba-1 expression in sham-operated animals.** Iba-1 expression in striatum of non-diabetic controls (sham non-T2D), diabetic mice (sham T2D-VH) and diabetic mice treated daily with 10 mg/kg Empagliflozin (sham T2D-E). N = 4-5 per group. Data are presented as mean ± SD. Statistical significance was calculated using Brown-Forsythe and Welch one way ANOVA followed by a *post hoc* multiple comparisons test using the two-stage step-up method of Benjamini, Krieger and Yekutieli. Results were considered significant if p < 0.05. * denotes p < 0.05.

Non-T2D

T2D-VH

T2D-E

**Fig S3: Effect of Empagliflozin on pericytes activation.** Evaluation in the striatum of non-diabetic controls (non-T2D), diabetic mice (T2D-VH) and diabetic mice treated with Empagliflozin (T2D-E) after stroke of pericyte activation, calculated as density of the colocalizing NG2 and CD13 positive signals normalized to total CD13 density. Data are presented as mean ± SD. Statistical significance was calculated using two-way ANOVA followed by Benjamini, Krieger and Yekutieli multiple comparisons test. Results were considered statistically significant if p < 0.05. p values are indicated between relevant. Scale bar = 50 μm. non-T2D n = 5, T2D-VH n = 5, T2D-E n = 6.

 **Fig S4: Effect of Empagliflozin on BBB leakage and vascularization after stroke.** Expression in the striatum of non-diabetic controls (non-T2D), diabetic mice (T2D-VH) and diabetic mice treated with Empagliflozin (T2D-E) after stroke of albumin (A) and fibrinogen (B) to evaluate BBB leakage; and other parameters related to vascularization such as vessels total length (C), branches number (D) and length (E). Data are presented as mean ± SD. Statistical significance was calculated using two-way ANOVA followed by Benjamini, Krieger and Yekutieli multiple comparisons test. Results were considered statistically significant if p < 0.05. p values are indicated between relevant. non-T2D n = 5, T2D-VH n = 5, T2D-E n = 6.

**Fig S5:** **Effect of Empagliflozin on vascularization and BBB leakage in sham-operated animals.** Expression in the striatum of non-diabetic controls (non-T2D), diabetic mice (T2D-VH) and diabetic mice treated with Empagliflozin (T2D-E) after sham-operation of pericyte density (A), vessels density (B), pericyte coverage (C), parenchymal pericyte density (D), activated pericytes (E), vessels length (F) branches number (G) and length (H) and BBB leakage (I, L). Data are presented as mean ± SD. Statistical significance was calculated using one-way ANOVA followed by Benjamini, Krieger and Yekutieli multiple comparisons test. Results were considered statistically significant if p < 0.05. p values are indicated between relevant comparisons. non-T2D n = 5, T2D-VH n = 5, T2D-E n = 5.
